# SGLT2 Inhibition Induces Cardioprotection by Increasing Parasympathetic Activity

**DOI:** 10.1161/CIRCRESAHA.124.324708

**Published:** 2024-12-17

**Authors:** Maryna V. Basalay, Alla Korsak, Zhenhe He, Alexander V. Gourine, Sean M. Davidson, Derek M. Yellon

**Affiliations:** 1 The Hatter Cardiovascular Institute, University College London, United Kingdom (M.V.B., Z.H., S.M.D., D.M.Y.).; 2 Department of Neuroscience, Physiology and Pharmacology, Centre for Cardiovascular and Metabolic Neuroscience, University College London, United Kingdom (A.K., A.V.G.).

**Keywords:** glucose, heart failure, myocardial infarction, sodium-glucose transporter 2, sodium-glucose transporter 2 inhibitors, vagus nerve

**Meet the First Author, see p 160**

Inhibitors of SGLT2 (sodium-glucose cotransporter-2) are used in the treatment of type 2 diabetes. In addition to their glucose-lowering effect, SGLT2 inhibitors exhibit significant cardioprotection and renoprotection. Large-scale clinical trials demonstrated benefits of SGLT2 inhibitors in patients with heart failure with either reduced or preserved ejection fraction.^1^ However, the mechanisms underlying the beneficial cardiovascular effects of SGLT2 inhibitors are not entirely clear as SGLT2 is not expressed in the heart and SGLT2 inhibitors are beneficial irrespective of diabetes. Moreover, evidence suggests that these inhibitors may induce cardioprotection independently of SGLT2.^2^

Autonomic dysfunction characterized by sympathetic activation and parasympathetic (vagal) withdrawal accelerates the development and progression of cardiovascular disease. We hypothesized that improved autonomic balance and increased parasympathetic activity may be responsible for the beneficial effects of SGLT2 inhibitors. This study conducted in rats was designed to test this hypothesis.

The experiments were performed in accordance with the UK Animals (Scientific Procedures) Act (1986) with project approval from the UCL Animal Care and Use Committee. Male Sprague-Dawley rats (150–175 g) were given the SGLT2 inhibitor ertugliflozin in their diet (5 or 20 mg/kg per day for 3–7 days), and the effect of this treatment on the activity of vagal preganglionic neurons (as a measure of parasympathetic activity) and the extent of ischemia/reperfusion injury following myocardial infarction were evaluated. Glycosuria was observed in animals given ertugliflozin confirming the efficacy of treatment.

For the neuronal recordings, rats were anesthetized with urethane (induction, 1.3 g/kg, IP; maintenance, 10–25 mg/kg per hour IV) and instrumented.^3^ The electrical activity (action potential firing) of neurons in the dorsal vagal motor nucleus was recorded using carbon fiber microelectrodes (Figure [A and B]). Vagal neurons were identified by antidromic activation in response to electrical stimulation of the cervical vagus nerve. The mean firing rate of vagal neurons recorded in rats treated with ertugliflozin was markedly higher than in animals kept on a control diet: 8.6±1.8 Hz average discharge rate recorded from 27 neurons in 5 animals treated with 5 mg/kg per day ertugliflozin (*P*=0.038) and 9.7±1.7 Hz (38 neurons) recorded in 6 animals treated with 20 mg/kg per day ertugliflozin (*P*=0.006) versus 3.1±0.8 Hz (39 neurons) recorded in 6 rats on a control diet (Figure [C]). These data provide direct neurophysiological evidence that ertugliflozin treatment increases vagal activity. A decrease in resting heart rate was recorded in rats treated with ertugliflozin (329±5 versus 346±5 bpm in controls; *P*=0.020; n=34, 33).

**Figure.**
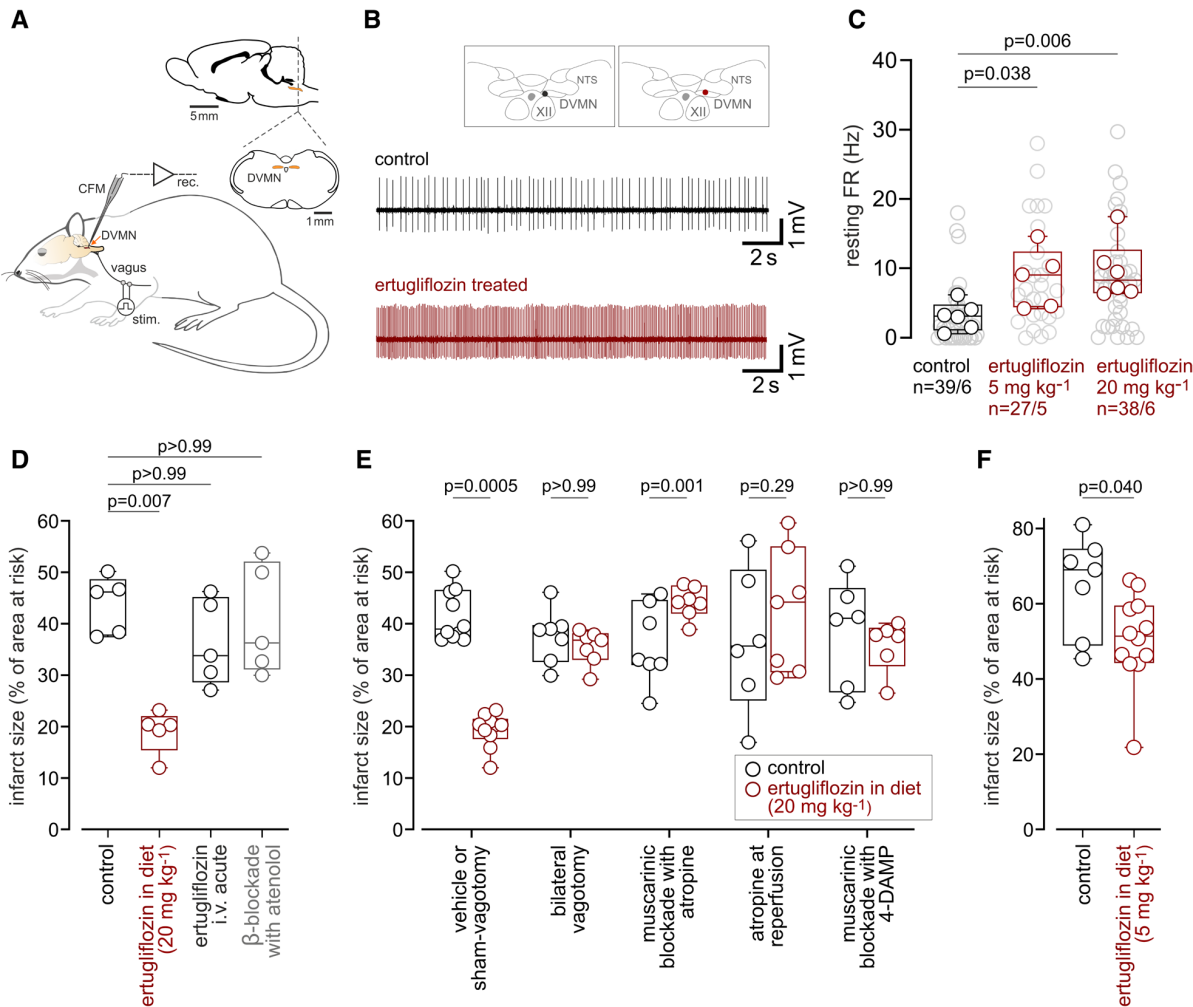
**SGLT2 inhibitor ertugliflozin induces cardioprotection via an increase in vagal parasympathetic activity. A**, Anesthetized rats instrumented for recording (rec) vagal preganglionic neurons in the dorsal vagal motor nucleus (DVMN) using carbon fiber microelectrodes (CFMs; see the Major Resources Table in the Data Supplement). Vagal neurons were identified by antidromic activation in response to vagus nerve stimulation (stim.). **B**, Examples of action potential firing illustrating DVMN neuron activity in control or ertugliflozin-treated rats. **C**, Resting action potential firing rate (FR) of DVMN neurons in control rats (n=6) or those treated with 5 (n=5) or 20 (n=6) mg/kg per day ertugliflozin. Individual neuron FR (light gray) and mean FR of DVMN neurons per animal are shown. *P* values: Kruskal-Wallis with Dunn post hoc. **D**, Chronic (in diet) but not acute (30 minutes before ischemia IV) administration of ertugliflozin is cardioprotective. β-Blockade with atenolol (10 minutes before ischemia) had no effect on infarct size in control rats. n=5 per group. *P* values: Kruskal-Wallis with Dunn post hoc. **E**, Bilateral vagotomy or systemic muscarinic receptor blockade abolishes cardioprotection induced by SGLT2 inhibition. Rats were subjected to sham vagotomy (n=8), bilateral vagotomy (n=7), intravenous infusion of atropine 10 minutes before myocardial ischemia (n=7), atropine 5 minutes before the onset of reperfusion (n=7), or M3 muscarinic receptor antagonist 4-DAMP (1,1-dimethyl-4-diphenylacetoxypiperidinium iodide) administered intravenously 10 minutes before myocardial ischemia (n=6). *P* values: aligned rank transform ANOVA with pairwise comparisons and Bonferroni correction. **F**, Cardioprotection induced by 5 mg/kg per day ertugliflozin (n=7, 12). *P* value: Mann-Whitney *U* test. Median is shown, with upper/lower quartiles and range. n indicates individual neurons recorded/number of animals per experimental group; and NTS, nucleus tractus solitarius.

Vagal activity limits ischemia/reperfusion injury following myocardial infarction.^4^ If treatment with ertugliflozin increases vagal activity, it would also be expected to be cardioprotective. We used an established model of myocardial ischemia/reperfusion injury in rats anesthetized with isoflurane.^5^ We evaluated the effect of ertugliflozin on myocardial infarction developing after 40 minutes of coronary artery occlusion followed by 2 hours of reperfusion.

The average infarct size in rats treated with ertugliflozin was significantly smaller compared with that in rats maintained on a control diet (19±2% versus 43±3%; *P*=0.007; Figure [D]). Acute administration of ertugliflozin (1 mg/kg IV) 30 minutes before myocardial ischemia had no effect on infarct size in rats kept on control diet (Figure [D]), indicating that ertugliflozin-induced cardioprotection requires prolonged administration of the drug. β-Blockade (atenolol) had no effect on infarct size in this model (Figure [D]). If increased vagal activity is responsible for cardioprotection induced by ertugliflozin, then blockade of vagal influences would be expected to prevent the infarct-limiting effects of the drug. Bilateral sectioning of vagi at the cervical level or systemic treatment with muscarinic antagonists atropine (2 mg/kg IV) or 4-DAMP (1,1-dimethyl-4-diphenylacetoxypiperidinium iodide) (2 mg/kg IV) abolished ertugliflozin cardioprotection (infarct sizes, 35±1% after vagotomy; 44±1% and 36±2% under conditions of muscarinic blockade with atropine and 4-DAMP, respectively; Figure [E]). A lower dose of 5 mg/kg per day ertugliflozin also exerted significant cardioprotection, although this effect was less pronounced than that of 20 mg/kg per day ertugliflozin (Figure [F]).

These data suggest that increases in vagal parasympathetic activity underlie cardioprotection induced by SGLT2 inhibitors. First, treatment with ertugliflozin increases (by >3-fold) the activity of the key population of vagal neurons. The effect of ertugliflozin on vagal activity exceeded that of exercise training,^3^ a known positive intervention for vagal modulation. Second, ertugliflozin induces potent cardioprotection against ischemia/reperfusion injury, which is entirely dependent on the integrity of vagal pathways and cholinergic receptor–mediated signaling.

The established gold standard treatment of heart failure includes drugs that limit the sympathetic effects on the heart and kidneys (e.g., β-blockers). However, recruitment of vagal activity to redress autonomic balance as a treatment for heart failure has been more difficult to achieve. Patients on optimal therapy with persistent autonomic dysfunction have the worst prognosis. This study identifies SGLT2 inhibitors as pharmacological agents capable of increasing vagal tone, which may develop as a compensatory autonomic response to an altered metabolic state associated with urinary glucose loss. This effect underlies cardioprotection by SGLT2 inhibitors in the setting of ischemia/reperfusion, although other mechanisms may also contribute.

## Article Information

### Author Contributions

M.V. Basalay, A. Korsak, Z. He: conducted the experiments. A.V. Gourine, S.M. Davidson, D.M. Yellon: wrote the manuscript.

### Data Availability

Data will be available upon reasonable request.

### Sources of Funding

This study was supported by the British Heart Foundation (RG/19/5/34463).

### Disclosures

None.

## Supplementary Material
