# Supplementary material for: SGLT2 Inhibition Induces Cardioprotection by Increasing Parasympathetic Activity
Source: Circ Res. 2024 Dec 17;136(2):229–31. doi: 10.1161/CIRCRESAHA.124.324708 (PMC11741129; doi:10.1161/CIRCRESAHA.124.324708)
Supplement: Supplementary file 1 [file res-136-229-s001.pdf]

Major Resources Table

Animals

Species	Vendor or Source	Background Strain	Sex	Persistent ID / URL
Rattus norvegicus	Charles River	Sprague Dawley	male	https://emodels.criver.com/product/001

Other

Description	Source / Repository	Persistent ID / URL
Ertugliflozin	MedChemExpress Europe	Cat. HY-15461
Atenolol	Sigma-Aldrich	Cat. A7655
Atropine	Sigma-Aldrich	Cat. a0257
4-DAMP	Tocris	Cat. 048216
Urethane	Sigma-Aldrich	Cat. U2500
Carbon fibre microelectrodes	Kation Scientific	Cat. E1011-7

1. Hemodynamic data prior to ischemia, for experimental groups related to Panel D of the Main Figure

**Mean BP**	**Mean**	**SEM**	**n**
**Control**	69	3	5
**Ertugliflozin in diet**	72	4	5
**Ertugliflozin acute**	73	2	5
**b-blockade with atenolol**	68	3	5

No significant differences.

**HR**	**Mean**	**SEM**	**n**
**Control**	372	7	5
**Ertugliflozin in diet**	356	13	5
**Ertugliflozin acute**	327	16	5
**b-blockade with atenolol**	309*	12	5

Control vs atenolol IV: P=0.01

2. Hemodynamic data prior to ischemia, for experimental groups related to Panel E of the Main Figure

**Mean BP**	**Mean**	**SEM**	**n**
**Vehicle**	70	2	10
**Vehicle + Ertugliflozin**	76	4	10
**Bilateral vagotomy**	81	6	7
**Bilateral vagotomy + Ertugliflozin**	77	5	7
**Atropine**	75	4	7
**Atropine + Ertugliflozin**	75	5	7
**Atropine at Reperfusion**	73	2	6
**Atropine at R + Ertugliflozin**	75	4	7
**4-DAMP**	71	3	6
**4DAMP + Ertugliflozin**	71	2	6

No significant differences.

DOI [to be added]

HR	Mean	SEM	n
**Vehicle**	352	10	10
**Vehicle + Ertugliflozin**	335	10	10
**Bilateral vagotomy**	378*	7	7
**Bilateral vagotomy + Ertugliflozin**	381[x]	7	7
**Atropine**	356	13	7
**Atropine + Ertugliflozin**	353	7	7
**Atropine at Reperfusion**	339	9	6
**Atropine at R + Ertugliflozin**	340	9	7
**4-DAMP**	362	9	6
**4DAMP + Ertugliflozin**	339	4	6

*P=0.04 Vagotomy vs Ertu

[x] = 0.02 Ertu + Vagotomy vs Ertu

3. Hemodynamic data prior to ischemia, for experimental groups related to Panel F of the Main Figure

Mean BP	Mean	SEM	n
**Control**	123	5	7
**Ertugliflozin in diet 5 mkg/kg**	111	5	12

No significant differences.

HR	Mean	SEM	n
**Control**	398	13	7
**Ertugliflozin in diet 5 mkg/kg**	357	10	12

Ertu vs Control P = 0.02

DOI [to be added]

ARRIVE

The ARRIVE guidelines 2.0: author checklist

The ARRIVE Essential 10

These items are the basic minimum to include in a manuscript. Without this information, readers and reviewers cannot assess the reliability of the findings.

Item		Recommendation	Section/line number, or reason for not reporting
Study design	1	For each experiment, provide brief details of study design including:	
		a. The groups being compared, including control groups. If no control group has been used, the rationale should be stated.	p2line22,36, figure legend.
		b. The experimental unit (e.g. a single animal, litter, or cage of animals).	Major Resource table
Sample size	2	a. Specify the exact number of experimental units allocated to each group, and the total number in each experiment. Also indicate the total number of animals used.	figure legend
		b. Explain how the sample size was decided. Provide details of any *a priori* sample size calculation, if done.	Major Resource table
Inclusion and exclusion criteria	3	a. Describe any criteria used for including and excluding animals (or experimental units) during the experiment, and data points during the analysis. Specify if these criteria were established *a priori*. If no criteria were set, state this explicitly.	Major Resource table
		b. For each experimental group, report any animals, experimental units or data points not included in the analysis and explain why. If there were no exclusions, state so.	Major Resource table
		c. For each analysis, report the exact value of *n* in each experimental group.	figure legend
Randomisation	4	a. State whether randomisation was used to allocate experimental units to control and treatment groups. If done, provide the method used to generate the randomisation sequence.	Major Resource table
		b. Describe the strategy used to minimise potential confounders such as the order of treatments and measurements, or animal/cage location. If confounders were not controlled, state this explicitly.	Major Resource table
Blinding	5	Describe who was aware of the group allocation at the different stages of the experiment (during the allocation, the conduct of the experiment, the outcome assessment, and the data analysis).	Major Resource table
Outcome measures	6	a. Clearly define all outcome measures assessed (e.g. cell death, molecular markers, or behavioural changes).	p2line31-32, supp info
		b. For hypothesis-testing studies, specify the primary outcome measure, i.e. the outcome measure that was used to determine the sample size.	Major Resource table
Statistical methods	7	a. Provide details of the statistical methods used for each analysis, including software used.	Major Resource table, figure legend.
		b. Describe any methods used to assess whether the data met the assumptions of the statistical approach, and what was done if the assumptions were not met.	Major Resource table
Experimental animals	8	a. Provide species-appropriate details of the animals used, including species, strain and substrain, sex, age or developmental stage, and, if relevant, weight.	Major Resources Table
		b. Provide further relevant information on the provenance of animals, health/immune status, genetic modification status, genotype, and any previous procedures.	Major Resources Table
Experimental procedures	9	For each experimental group, including controls, describe the procedures in enough detail to allow others to replicate them, including:	p2line22,29-34,47-49
		a. What was done, how it was done and what was used.	p2line23,p3line1
		b. When and how often.	p2line20
		c. Where (including detail of any acclimatisation periods).	
		d. Why (provide rationale for procedures).	p2line15-17
Results	10	For each experiment conducted, including independent replications, report:	
		a. Summary/descriptive statistics for each experimental group, with a measure of variability where applicable (e.g. mean and SD, or median and range).	p2line36-43,p3line5, 14-15,figure legend
		b. If applicable, the effect size with a confidence interval.	n/a

DOI [to be added]

The Recommended Set

These items complement the Essential 10 and add important context to the study. Reporting the items in both sets represents best practice.

Item		Recommendation	Section/line number, or reason for not reporting
Abstract	11	Provide an accurate summary of the research objectives, animal species, strain and sex, key methods, principal findings, and study conclusions.	no abstract in short communication
Background	12	a. Include sufficient scientific background to understand the rationale and context for the study, and explain the experimental approach.	p2line2-17
		b. Explain how the animal species and model used address the scientific objectives and, where appropriate, the relevance to human biology.	insufficient space available to report this in short communication format
Objectives	13	Clearly describe the research question, research objectives and, where appropriate, specific hypotheses being tested.	p2line13-17
Ethical statement	14	Provide the name of the ethical review committee or equivalent that has approved the use of animals in this study, and any relevant licence or protocol numbers (if applicable). If ethical approval was not sought or granted, provide a justification.	UK Home Office licence PP9987686
Housing and husbandry	15	Provide details of housing and husbandry conditions, including any environmental enrichment.	Major Resource table
Animal care and monitoring	16	a. Describe any interventions or steps taken in the experimental protocols to reduce pain, suffering and distress.	Major Resource table
		b. Report any expected or unexpected adverse events.	Major Resource table
		c. Describe the humane endpoints established for the study, the signs that were monitored and the frequency of monitoring. If the study did not have humane endpoints, state this.	Major Resource table
Interpretation/ scientific implications	17	a. Interpret the results, taking into account the study objectives and hypotheses, current theory and other relevant studies in the literature.	p3line20-25
		b. Comment on the study limitations including potential sources of bias, limitations of the animal model, and imprecision associated with the results.	insufficient space available to report this in short communication format
Generalisability/ translation	18	Comment on whether, and how, the findings of this study are likely to generalise to other species or experimental conditions, including any relevance to human biology (where appropriate).	p3line31-33
Protocol registration	19	Provide a statement indicating whether a protocol (including the research question, key design features, and analysis plan) was prepared before the study, and if and where this protocol was registered.	Major Resource table
Data access	20	Provide a statement describing if and where study data are available.	Major Resource table
Declaration of interests	21	a. Declare any potential conflicts of interest, including financial and non-financial. If none exist, this should be stated.	p3line46
		b. List all funding sources (including grant identifier) and the role of the funder(s) in the design, analysis and reporting of the study.	p3line39-45

Animal husbandry

All rats were kept in standard laboratory cages with bedding and maintained on a 14:10-hour light/dark cycle. The housing room was maintained at 22°C] with relative humidity around 55% (+-10%). Rats were provided ad libitum access to standard chow and water. A one-week acclimatization period was provided to allow animals to adjust to the new environment. Rats were housed in groups of 4 per cage to minimize stress and promote social interaction. Animals were observed daily during treatment for any expected or unexpected adverse events and none were observed.

Experimental design and data

The experimental design was pre-defined but was not pre-registered. Each animal was considered one experimental unit. Sample sizes were determined based on prior experience with this type of experiment in our laboratory, and expected effect size, although an priori power calculation was not performed. Post hoc power calculations

DOI [to be added]

demonstrated that the experiments were sufficiently well powered. A priori exclusion criteria included any animal death prior to the end of the experiment,

Animal were allocated randomly to each treatment group using https://www.randomizer.org/ No specific strategies were used to minimize potential confounders. No animals were excluded from our analysis. The experimenter was non blinded to the experimental intervention, but the analysis of heart infarct size was conducted blinded to treatment group. Experimental data is available from the corresponding author on reasonable request.

Ischaemia and reperfusion, and infarct size measurement

The rats were intubated, their right common carotid artery and left jugular vein cannulated, and the chest was opened by dissecting two ribs to the left of the sternum. The heart was then exposed using a chest retractor. After a 10-min stabilization period, the arterial blood was collected into a capillary tube to measure blood pH. If required, the parameters of ventilation were adjusted, with a re-check of the pH after 10 min. The left anterior descending coronary artery (LAD) was then ligated with a polypropylene suture, needle size 5–0, for 30 min after which it was reperfused for 2 h. Cling film was used to cover the opening of the chest throughout the experiment to prevent cooling and drying of the heart. Blood pressure and heart rate were recorded throughout the experiment, and body temperature maintained at 36.4 to 37.5 °C. At the end of the reperfusion period, the LAD was reoccluded and the hearts perfused, via the jugular vein, with 5% Evan's blue dye to delineate the area at risk (AAR). The heart was then rapidly excised, the right ventricle removed, and the left ventricle frozen at −80 °C and sliced into 6–7 slices of equal thickness. Each slice was scanned from both sides to obtain the images of the AAR. After that, the infarcted myocardium was detected by incubating the frozen heart slices with 1% 2,3,5-triphenyltetrazolium chloride in Tris buffer (pH 7.4) for 15 min at 37 °C and fixing in 4% formalin for 24 h. Finally, the slices were scanned, again from both sides, and each slice was weighed. The AAR and infarct size (IS) were planimetrically evaluated using ImageJ (https://imagej.nih.gov/ij/). The weight of the AAR was normalized to the weight of the left ventricle. IS was expressed as a proportion of the weight of the infarcted myocardium to

the weight of the AAR. None of the animals died prior to completion of the protocol nor were any excluded. AAR was comparable in all the experimental groups. The primary outcome measure was infarct size / AAR.

Analysis

Graphpad prism v10.2.2 was used for statistical analysis. The infarct data was assumed to be normally distributed, based on extensive prior experience. The neuron firing data was not assumed to be normally distributed. As such it was analyzed using non-parametric approach as described in figure legend.

DOI [to be added]
